# Natural Killer Cell Interactions with Classical and Non-Classical Human Leukocyte Antigen Class I in HIV-1 Infection

**DOI:** 10.3389/fimmu.2017.01496

**Published:** 2017-11-14

**Authors:** Angelique Hölzemer, Wilfredo F. Garcia-Beltran, Marcus Altfeld

**Affiliations:** ^1^First Department of Internal Medicine, University Medical Center Hamburg-Eppendorf, Hamburg, Germany; ^2^German Center for Infection Research (DZIF), Partner site Hamburg-Lübeck-Borstel-Riems, Hamburg, Germany; ^3^Harvard Medical School, Boston, MA, United States; ^4^Institute for Immunology, University Medical Center Hamburg-Eppendorf, Hamburg, Germany

**Keywords:** HIV-1, innate immunity, natural killer cells, killer cell immunoglobulin-like receptor, human leukocyte antigen class I, human leukocyte antigen-F, human leukocyte antigen-E

## Abstract

Natural killer (NK) cells are effector lymphocytes of the innate immune system that are able to mount a multifaceted antiviral response within hours following infection. This is achieved through an array of cell surface receptors surveilling host cells for alterations in human leukocyte antigen class I (HLA-I) expression and other ligands as signs of viral infection, malignant transformation, and cellular stress. This interaction between HLA-I ligands and NK-cell receptor is not only important for recognition of diseased cells but also mediates tuning of NK-cell-effector functions. HIV-1 alters the expression of HLA-I ligands on infected cells, rendering them susceptible to NK cell-mediated killing. However, over the past years, various HIV-1 evasion strategies have been discovered to target NK-cell-receptor ligands and allow the virus to escape from NK cell-mediated immunity. While studies have been mainly focusing on the role of polymorphic HLA-A, -B, and -C molecules, less is known about how HIV-1 affects the more conserved, non-classical HLA-I molecules HLA-E, -G, and -F. In this review, we will focus on the recent progress in understanding the role of non-classical HLA-I ligands in NK cell-mediated recognition of HIV-1-infected cells.

## Introduction

Untreated HIV-1 infection will lead to progressive, severe, and mostly fatal immune deficiency in the vast majority of individuals. Protective HIV-1 immunity is observed in a small subset of subjects whose immune system can naturally control HIV-1 infection and who are termed “elite controllers.” Despite intense research in this area over the past decades, the correlates leading to protective immunity are still insufficiently understood. Host genetics alone can only explain approximately 20% of the variable outcomes between individuals observed in the natural course of infection (1). Nonetheless, a consistently documented key genetic determinant of HIV-1 control is the presence of particular human leukocyte antigen (HLA) class I alleles. This strong association between classical HLA-I alleles and HIV-1 disease outcome has been identified in genome-wide association studies (1, 2) as well as in large cohorts studying the immunogenetics of HIV-1 disease (3, 4). The protective effects of certain HLA-I alleles have mostly been attributed to enhanced CD8^+^ T lymphocyte-mediated immunity (5–7). HLA-I presentation of HIV-1 epitopes derived from conserved sequences of HIV-1 to CD8^+^ T cells can pressure the virus to select for mutations in these epitopes, but viral escape can be associated with costs in viral fitness (8). Indeed, early CD8^+^ T-lymphocyte responses contribute to the initial drop in HIV-1 peak viremia and with this, first HIV-1 escape mutations arise (9). Other protective factors in HIV-1 infection include enhanced proliferation potential of T lymphocytes (10, 11), polyfunctional immune responses (12, 13), variations in host restriction factors (14), and variants in HIV-1 coreceptors, in particular, of CCR5 (15, 16).

Over the past years, the role of antiviral innate immune responses mediated by natural killer (NK) cells in HIV-1 infection has been increasingly appreciated (17, 18). *In vitro*, NK cells can inhibit HIV-1 replication in autologous CD4^+^ T cells as effectively as CD8^+^ T cells (19). Additionally, the strong protective effect of host HLA-I alleles on disease progression has been linked to receptor families recognizing HLA-I. These include killer-cell immunoglobulin-like receptors (KIRs), predominantly expressed on NK cells (20), and leukocyte immunoglobulin-like receptors (LILRs), expressed on professional antigen presenting cells such as dendritic cells (DCs), monocytes, macrophages, and B cells, but also on T cells and NK cells (21). Indeed, accumulating data from population studies have identified certain *KIR, LILR*, and *HLA-I* allele combinations associated with slower HIV-1 disease progression (22–24), which has helped decipher a further piece of complex host genetics in HIV-1 disease variability.

Natural killer cells comprise 5–15% of the circulating lymphocytes (25) and their role in controlling viral infections has been long established (26). Two major subsets exist: CD56^bright^CD16^dim/neg^ and CD56^dim^CD16^pos^ NK cells (25). These differ in their expression of key NK-cell receptors, response to soluble factors and cellular targets, capacity for cytotoxicity, and production of immunomodulatory cytokines (27). NK cells are a crucial first line of defense that detect infected cells before antigen sensitization has occurred (28, 29), and therefore, they precede adaptive immunity in the early phases of HIV-1 infection. Indeed, there is evidence that the early events following infection prior to the development of a specific immune response can determine the viral set point and influence the clinical course of infection (30). In acute HIV-1 infection, a rapid expansion occurs in predominantly cytotoxic CD56^dim^ NK cells, prior to CD8^+^ T cell expansion (31). On the other hand, in chronic HIV-1 infection, a redistribution of NK cells toward less functional subsets can be observed (32–35) and the presence of persistent viremia appears to deteriorate NK-cell function (19, 34, 36). Overall, the full extent of receptor-ligand interactions between NK cells and HIV-1–infected target cells in HIV-1 infection leading to either NK-cell expansion/killing or exhaustion is highly complex and not yet fully understood.

Natural killer cells, as members of the innate immune system, express a plethora of germline-encoded receptors, and their effector function is determined by integration of inhibitory and activating NK-cell receptor signaling, whereby inhibitory signals tend to be dominant (27). Major NK-cell receptor families are (i) natural cytotoxicity receptors (i.e., NKp46, NKp44, and NKp30), which deliver mainly activating signals, (ii) the KIR family, encompassing inhibitory and activating members and monitoring HLA-I, (iii) the C-type lectins with activating natural killer group 2D (NKG2D) and the heterodimers NKG2A-CD94 and NKG2C-CD94, and (iv) the FcγRIIIa receptor (CD16), which can bind to the Fc-region of IgG antibodies. Critical activating signals can also be delivered by other coreceptors including 2B4, DNAM-1, or CD2 (37, 38). Differential expression of activating and inhibitory receptors allows for a certain degree of specificity and shaping of NK-cell function in response to different stimuli. Ultimately, the stochastic expression of receptors on each NK cell leads to substantial NK-cell diversity and determines the differential response to target cells (39, 40).

HIV-1–infected cells can become vulnerable to NK cell-mediated killing by upregulation of stress signals recognized by activating NK-cell receptors and/or by downregulation of inhibitory NK-cell-receptor ligands. Of note, signaling *via* the FcγRIIIa receptor (CD16), which mediates antibody-dependent cellular cytotoxicity (ADCC), is sufficient to induce NK-cell activation on its own (37). However, the strength of CD16-mediated activation is dependent on tuning of NK-cell responsiveness through inhibitory interactions of KIR or NKG2A with HLA class I (41, 42). Stress ligands upregulated on HIV-1–infected cells are the major histocompatibility complex (MHC) class-I-chain-related proteins (MIC-) A and -B, the UL16-binding proteins (ULBPs) 1–3, which are the ligands for the activating NKG2D receptors (43, 44), and a yet unknown ligand for NKp44 (45, 46). In turn, HIV-1 encodes for multiple accessory proteins with pleiotropic functions to overcome host restriction factors and host immune responses (47–49). The upregulation of stress ligands such as ULBPs and MIC-A/B is counteracted *via* HIV-1 Nef (50) and the ligands for coactivating receptors such as NTB-A and DNAM-1 are downregulated *via* HIV-1 Vpu and partially Nef (51–53). The impact of HIV-1 Nef and Vpu on HLA class I expression will be discussed later. In this review, we will focus on the recent progress in understanding the interplay of HLA-I with HLA-I binding NK-cell receptors, and how this interaction either limits HIV-1 replication or is exploited by the virus to enhance pathogenesis.

### KIR–HLA Interactions in HIV-1 Disease Progression and Acquisition

Classical and non-classical HLA-I genes (also known as HLA-Ia and HLA-Ib, respectively) are located within the MHC region p21.3 on chromosome 6, the most polymorphic region of the human genome. An extensive amount of allelic variation occurs within the region encoding for classical HLA-I genes (54). In contrast, non-classical HLA-I alleles display varying degrees of oligomorphism. To date, the classical *HLA-A, HLA-B*, and *HLA-C* loci comprise >10,000 alleles encoding for 8,662 distinct proteins, whereas the non-classical *HLA-E, HLA-F*, and *HLA-G* loci combined encode for 101 alleles and only 30 proteins (The Immuno Polymorphism Database, as of July 2017) (55, 56).

Classical HLA-I is ubiquitously expressed on nucleated cells. Given that the primary function of HLA-I is to present peptides derived from degradation of intracellular proteins (57), it is not surprising that variations mainly occur in regions surrounding the peptide-binding groove (58) so as to maximize diversity of peptides presented across different gene and allele products. Under pathologic conditions such as malignant transformation or infection with intracellular pathogens, HLA-I presents antigenic peptides and thereby can elicit an immune response *via* HLA-I restricted cytotoxic CD8^+^-T cells. Historically, it was thought that NK cells only respond to changes in surface levels of classical HLA class I [to missing-self (59)], but there is increasing evidence that KIR can bind differentially depending on the HLA-class I presented peptide (60–65).

In 2007, the first genome-wide association studies reported three protective single-nucleotide polymorphisms (SNPs) in HIV-1 disease (2). The presence of these SNPs was associated with lower viral set point in chronically HIV-1–infected subjects and together explained almost 15% of interindividual disease variability. Strikingly, all three SNPs were located in the MHC region of chromosome 6, emphasizing the crucial role of HLA class I in HIV-1 infection. The first SNP is in high linkage disequilibrium with HLA-B*57, a second SNP was located 35bp upstream of the HLA-C locus, and results in higher HLA-C expression levels. The last SNP was linked to an RNA polymerase subunit, ZNRD1 and affected the time to AIDS progression. Subsequent genome-wide association studies confirmed the first two SNPs and identified six additional SNPs associated with HIV-1 disease control in two different ethnic cohorts. Again, all SNPs were concentrated around the HLA-I region (1). Accordingly, the strongest HLA class I protective effects so far are reported for HLA-B*57 (66, 67) and HLA-B*27 (4, 68); two HLA class I alleles carrying the serologically defined Bw4 motif (determined by the amino acids 77–83). There is a strong association of HLA-Bw4 homozygosity with the ability to suppress viral replication of HIV-1 and with delayed time to AIDS progression (69).

The genes encoding for KIRs are located within the leukocyte receptor cluster on Chromosome 19q13.4, which additionally encodes Ig-like transcripts (ILTs) [also termed leukocyte Ig-like receptors (LIRs)], and leukocyte-associated inhibitory receptors (70). The KIR locus exhibits substantial polymorphism, in its degree only second to the MHC region in the human genome (71). KIRs can be subdivided into two different classes: KIRs with two extracellular Ig-like domains (KIR2Ds) and those with three domains (KIR3Ds). These Ig-like domains are classified as D0, D1, or D2. Type 1 KIR2Ds contain a D1 domain distal to a D2 domain, type 2 KIR2Ds (KIR2DL4 and KIR2DL5) have a D0–D2 domain organization, and KIR3Ds have all three domains as D0–D1–D2. In general, KIR2Ds bind to HLA-C and KIR3D bind to HLA-A and B-ligands (72, 73). Regarding signaling capacity, a long cytoplasmic tail (KIR-L) renders the KIR inhibitory as it contains immune tyrosine inhibitory motifs (ITIMs), whereas a short cytoplasmic tail (KIR-S) associates to adaptor molecules such as DAP12 and delivers activating signals (74). An exception to this is KIR2DL4, which holds an ITIM in its long cytoplasmic tail, but also associates with activating adaptor elements (73). KIRs are a major receptor family on NK cells, but are also expressed on CD4^+^ and CD8^+^ T cells (both αβ and γδ T cells) (75–80). Of note, expression of inhibitory KIR on T cells is increased following chronic immune activation, as was observed in the case of CMV reactivation in a posttransplantation setting (81, 82). Increased KIR expression on bulk CD8^+^ T cells in HIV-1 infection has been reported, but barely detectable KIR expression was described, when investigating HIV-specific CD8^+^ T cells (83, 84). Overall, little is known about a modulation of KIR-expression on T cells with or without CMV reactivation in HIV-1–infected subjects.

### KIR3DS1/KIR3DL1 and HLA-Bw4^I80^

The first study associating KIRs to HIV-1 control came from the laboratory of Mary Carrington in 2002. This study showed that possessing *KIR3DS1* and an *HLA-B* allele with a Bw4 motif and an isoleucine at position 80 (HLA-Bw4^I80^) was associated with slower progression to AIDS, when compared to patients having only one or none of these alleles (22). A follow-up analysis by the same group reported a protective effect of combined KIR3DS1 and HLA-Bw4^I80^ against development of certain opportunistic infections in HIV-1–infected patients, also after controlling for presence of protective (e.g., HLA-B*57 and HLA-B*27) and deleterious (HLA-B*35) alleles (85). The *KIR3DS1/KIR3DL1* locus is unique in that it encodes functionally divergent alleles (86). The inhibitory KIR3DL1 binds to HLA-I allotypes that possess a Bw4 motif (HLA-Bw4, which can derive from *HLA-A* or *HLA-B* alleles). Polymorphisms in position 80 of these HLA-Bw4 molecules have been shown to modulate the strength of binding to KIR3DL1 (87, 88). In addition, the interaction of KIR3DL1 with HLA-Bw4 is sensitive to the sequence of the HLA-Bw4–presented peptide (61). Contrary to KIR3DL1, a ligand for its activating counterpart, KIR3DS1, remained initially unknown.

In a cohort of recently infected individuals, Barbour et al. did not detect a synergistic protective effect of *KIR3DS1* and HLA-Bw4^I80^ assessing viral load and CD4^+^ T cell loss. Nonetheless, encoding for at least one *KIR3DS1* allele was associated with higher CD4^+^ T cell counts and encoding for HLA-Bw4^I80^ alleles correlated with lower viral load, suggesting a protective, but independent effect of *KIR3DS1* and HLA-Bw4^I80^ (89). A further epidemiologic study reported that HIV-1 viral load at set point correlated positively with the number of *KIR3DS1* gene copies in the presence of HLA-B Bw4^I80^ ligands. Higher copy numbers of the *KIR3DL1* gene also correlated with lower viral set point in the presence of HLA-Bw4^I80^ and at least one copy of *KIR3DS1* (90). In addition, a study by Jiang et al. (91) in a Chinese cohort showed that *KIR3DS1/KIR3DL1* heterozygotes were enriched in HLA-Bw4^I80^–bearing long-term non-progressors with higher CD4^+^ T cell counts and decreased viral loads as compared to KIR3DL1 homozygotes or individuals without HLA-Bw4^I80^ (91).

As KIRs are predominantly expressed on NK cells, Martin et al.’s first report associating a KIR to an outcome in HIV-1 infection (22) triggered multiple studies on NK-cell functionality attempting to elucidate the underlying protective mechanism of *KIR3DS1* in combination with HLA-Bw4^I80^ in HIV-1 disease. In line with the epidemiological data, functional studies reported that NK cells derived from donors possessing *KIR3DS1* combined with HLA-Bw4^I80^ inhibited viral replication in infected autologous CD4^+^ T cells more potently than NK cells from donors having either or neither allele. Sorted KIR3DS1^+^ NK cells degranulated significantly more in response to HIV-1–infected HLA-Bw4–expressing CD4^+^ T cells compared to infected HLA-Bw6^+^ CD4^+^ T cells (92). A second study showed that NK cells from individuals encoding for *KIR3DS1* displayed enhanced cytotoxic function compared to NK cells from individuals without *KIR3DS1*, but this was independent of the presence of HLA-Bw4^I80^ (93). Also, in acutely HIV-1–infected subjects a preferential expansion of KIR3DS1^+^ NK cells—and to a lesser extent KIR3DL1^+^ NK cells—was observed, which persisted only in subjects bearing HLA-Bw4^I80^ (94). Morvan et al. reported an expansion of KIR3DS1^+^ NK cells in response to various non-specific stimuli, but KIR3DS1^+^ NK-cell function was not influenced by the presence of HLA-Bw4 in this setting. Nonetheless, the frequency of KIR3DS1^+^ NK cells and KIR3DS1 expression levels on NK cells were higher in healthy subjects with HLA-Bw4^I80^ than in those without HLA-Bw4^I80^ (95). Furthermore, HIV-1 viral inhibition assays demonstrated that in individuals encoding HLA-Bw4, having one copy of *KIR3DS1* and one or more copies of *KIR3DL1* resulted in increased antiviral capacity of bulk NK cells compared to individuals containing either *KIR3DS1* or *KIR3DL1* alone, which displayed the lowest amounts of viral inhibition (90). No differences were seen in HLA-Bw6 homozygous donors, whose NK cells had poor antiviral capacity. Having increasing copy numbers of *KIR3DL1* was correlated with elevated *KIR3DS1* transcript and frequency of KIR3DS1 expression on NK cells. Interestingly, this hinted at a *KIR3DL1*-related mechanism regulating the peripheral expansion and functionality of KIR3DS1^+^ NK cells (90). A more recent study reported that NK cells from *KIR3DS1* and HLA-Bw4^I80^ cocarriers produced higher levels of chemokines after cell contact with infected CD4^+^ T cells than NK cells derived from HLA-Bw6 homozygous donors, leading to superior inhibition of viral replication (96).

Understanding the mechanistic basis of the protective effect of *KIR3DS1* has proven difficult, as multiple attempts had failed to demonstrate a functional interaction of KIR3DS1 with its putative HLA-Bw4 ligand (74, 97) or for that matter, an interaction with any ligand. To add an additional layer of complexity, the combined genotype of high expressing KIR3DL1*h alleles and HLA-Bw4^I80^ (in particular HLA-B*57) conferred strong protection toward HIV-1 disease progression (23, 98). Indeed, increased target cell cytotoxicity was observed in NK cells derived from elite controllers with protective KIR3DL1*h/*y receptor genotypes along with its HLA-Bw4^I80^ ligand (99). As *KIR3DS1* homozygosity is rare, in the majority of studies investigating *KIR3DS1* and HLA-Bw4 epistasis, *KIR3DS1*-bearing subjects possessed also *KIR3DL1* as a potential confounding variable.

Protection by an inhibitory KIR in HIV-1 disease seems counterintuitive, but might be mediated through a process called NK-cell licensing or education. Expression of an inhibitory KIR during NK-cell development provides strong inhibitory signals in response to its specific HLA-I ligand, ensuring self-tolerance. This allows NK cells to acquire enhanced cytotoxic function, which becomes apparent once exposed to missing or altered self (100). KIR3DL1 allotypes indeed differ in their inhibition of NK-cell function, with an overall trend toward increasing inhibitory capacity in high-expressing KIR3DL1 allotypes (101). Thus, a potential explanation is that presence of high-expression KIR3DL1*h alleles together with HLA-Bw4^I80^ determines the increased cytotoxicity of KIR3DL1^+^ NK cells toward HIV-1–infected targets (taking into account that HLA-B is downregulated *via* actions of the HIV-1 accessory protein Nef) (23). Indeed, a study in slow progressors to AIDS reported increased polyfunctionality of NK cells from donors carrying the KIR3DL1*h/*y allele together with its HLA-B*57 ligand compared to HLA-Bw6 homozygous donors (102). Boudreau et al. recently demonstrated functionally that killing of HIV-1–infected targets *via* KIR3DL1^+^ NK-cells was dependent on the strength of NK-cell education *via* distinct combinations of KIR3DL1 and HLA-Bw4, with highest cytotoxicity mediated by high-expressing KIR3DL1 and HLA-Bw4^I80^ interactions (103). Moreover, NK cell education not only leads to enhanced functionality (104), but signaling through inhibitory KIRs on NK cells can additionally promote NK-cell survival (105), potentially leading to accumulation of educated NK cells expressing inhibitory receptors in chronic viral infection.

Supplementary evidence comes from studies in highly exposed HIV-1 seronegative individuals. One study reported a significant overrepresentation of *KIR3DS1* homozygosity in high-risk uninfected individuals compared to seroconverted individuals, independent of HLA-Bw4^I80^ (106). This group also reported an association of the KIR3DL1*h/*y-HLA-B*57 combined genotype with protection from HIV-1 acquisition (107). Another study showed enrichment of the HLA-Bw4 carrier–*KIR3DS1* homozygous genotype in HIV-1-exposed seronegative subjects (108). In summary, whereas the results from epidemiological studies are not clear-cut, these studies point toward a potential dual effect of *KIR3DS1* (with or without HLA-Bw4^I80^) on both the course of HIV-1 infection and HIV-1 acquisition.

### HLA-C and KIR2Ds

Genome-wide association studies have clearly implicated the *HLA-C* locus in HIV-1 control, identifying a protective SNP associated with higher HLA-C expression levels (1, 2). Interestingly, HLA-C surface expression levels are only 10% of surface levels of HLA-A and -B (109), and *HLA-C* alleles demonstrate less polymorphism compared to *HLA-B* (56). Nonetheless, individuals with high HLA-C expression levels have been shown to have a higher likelihood of mounting an HLA-C-restricted CD8^+^ T-cell response (110) and exhibit higher mutation rates in HLA-C–presented HIV-1 epitopes, indicating CD8^+^ T-cell pressure *via* HLA-C (111). However, given that virtually all individuals encode for KIRs (i.e., KIR2Ds) able to recognize cognate HLA-C molecules, it was proposed that NK cells might play an additional role in mediating the protective effect of higher HLA-C expression. Inhibitory KIR2DL1 binds to HLA-C group 2 allotypes (HLA-C2, which contain Asn77 and Lys80), whereas inhibitory KIR2DL2 and KIR2DL3, which are allelic products of the same *KIR2DL2/3* locus, bind to HLA-C group 1 allotypes (HLA-C1, which contain Ser77 and Asn80). Notably, KIR2DL3 also recognizes HLA-B*46:01 due to an intergenic miniconversion between HLA-B*15:01 and HLA-C*01:02 (65). It was long believed that while HIV-1 Nef downregulated HLA-A and HLA-B surface expression to avoid recognition by cytotoxic CD8^+^ T cells (112), it spared HLA-C surface expression to ensure inhibition of NK cells *via* engagement of KIR2DL. This paradigm—which initially arose from studies performed with lab-adapted HIV-1 strains—was recently revised, when Apps et al. demonstrated that HLA-C is downregulated by HIV-1 Vpu variants derived from most primary HIV-1 isolates. HIV-1 Vpu-mediated downregulation of HLA-C was shown to subsequently impair the ability of HLA-C–restricted CD8^+^ T cells to inhibit viral replication (113). Regarding NK-cell function, it was reported earlier that expression of HLA-C (and HLA-E) on activated, HIV-1–infected CD4^+^ T cells impaired NK-cell killing, whereas blocking the HLA-C interaction with KIR2D enhanced NK-cell cytotoxicity toward HIV-1–infected CD4^+^ T-cell blasts (114, 115). During primary HIV-1 infection, KIR2DL^+^ NK-cell frequencies increased with the presence of their cognate HLA-C ligand (e.g., KIR2DL1^+^ NK cells expanded in HLA-C2 homozygous individuals) and exhibited more polyfunctional responses, presumably due to a licensing effect (116). Downmodulation of HLA-C by various HIV-1 strains resulted in reduced binding of KIR2Ds to HIV-1–infected cells. Moreover, NK cells were able to sense alterations in HLA-C expression as measured by differing degrees of HIV-1-replication inhibition. Yet, remaining HLA-C surface levels were sufficient to inhibit antiviral function of licensed KIR2DL^+^ NK cells (encountering their cognate HLA-C ligand) compared to unlicensed NK cells (117). Thus, although NK cells licensed through inhibitory KIR2D exhibit increased functionality against HLA-I–deficient target cells, first reports indicate that this subset does not have superior antiviral function against HIV-1–infected targets expressing self-HLA-C.

### The Role of HIV-1 Peptides in KIR:HLA-I Interactions

HIV-1 exhibits an extraordinary ability to adapt to and evade host immune responses. The constant battle of the immune system attacking the virus and the virus evading leads to an extremely rapid accumulation of HIV-1 variants and quasispecies that, at least partially, escape from immune pressure (118, 119). Analyzing the major mechanisms of HIV-1 evasion and sites of sequence mutations provides direct insights into where the human immune system is able to apply critical pressure on the virus. A particular example is the rapid increase in HIV-1 mutations in HLA-I presented epitopes recognized by cytotoxic CD8^+^ T cells (CTL), which allows the virus to overcome adaptive immune pressure. These mutations can abrogate CTL recognition, but sometimes also impair viral replication (120). By now, a substantial body of evidence from structural (62, 63) and functional studies (60, 61, 65, 121–123) shows that KIR binding is modulated by the sequence of HLA-I–presented peptides, and in particular, C-terminal residues of these peptides. Unlike T cells, NK cells have germ-line encoded receptors that do not undergo recombination nor are they “specific” at discriminating self from non-self peptides (27). Instead, they have a moderate degree of peptide “sensitivity,” mediated in large part by KIR:HLA-I interactions, which allows NK cells to monitor for changes in the peptide repertoire expressed by target cells. In fact, common HIV-1 sequence variants can modulate binding of inhibitory KIR to HLA-I, and by this means modulate NK-cell function (61, 124, 125), which has also been demonstrated in the case of SIV (121). Alternatively, NK cells may respond to altered MHC-I peptide processing following induction of the immunoproteasome in response to viral infection. IFN-γ stimulation results in increased cleavage of peptides after hydrophobic and basic residues. Thereby, it alters the C-terminus of available peptides for HLA class I presentation [reviewed in Ref. (126)], which may ultimately affect KIR binding to HLA-I:peptide complexes presented on the cell surface of stressed cells.

Viral variants arising due to CTL-mediated pressure can in turn impact KIR recognition by (i) impairing binding to inhibitory KIRs (61, 127), (ii) reducing HLA-C surface levels (128), or (iii) enhancing binding to inhibitory KIRs directly, a mechanism termed as “double-escape” (129). Furthermore, several amino acid polymorphisms in the viral genome, which showed a significant enrichment in subjects having a specific *KIR* gene, have been identified (130). As one example, a polymorphism in the overlapping reading frame of *vpu* and *env* was associated with the presence of *KIR2DL2* in HIV-1–infected subjects. Antiviral activity of KIR2DL2^+^ NK cells against this viral variant was reduced *in vitro* (130). However, a role for HLA-I in this process could not be determined due to small sample size. A subsequent study in a larger cohort of HIV-1 clade C–infected individuals identified two viral sequence variants, that were significantly enriched in individuals in the presence of the combined KIR2DL3–HLA-C*03:04 genotype. One of the variants (T_gag303_V) was contained within a CTL epitope and located at the C-terminal end of the nonamer (YVDRFFKVL), but did not mediate escape from recognition by HLA-C*03:04-restricted CTLs compared to the wild-type sequence (131). This viral variant, however, enhanced binding to KIR2DL3 and inhibited KIR2DL3^+^ NK cells *in vitro* (132). Overall, these studies support the concept of KIR-mediated selection pressure on HIV-1 as an additional source driving viral evolution. Furthermore, a recent report showed that binding of KIR2DL2/3 to HLA-C1 allotypes is more selective to presented peptides than KIR2DL1 binding to HLA-C2 (60), further enhancing our mechanistic understanding of KIR:peptide:HLA-I interactions. Moreover, this study showed that certain peptides (including an HIV-1 Gag peptide) allow for binding of KIR2DLs to non-canonical HLA-C molecules (60). Taken together, whereas NK cells are not able to distinguish between self- and non-self peptides, KIR binding to HLA-I is certainly sensitive to changes in the peptide sequence presented on HLA-I molecules. This may in turn facilitate recognition of HIV-1–infected cells, potentially not only *via* presentation of viral peptides but also due to stressed-induced changes in the HLA-I-presented peptide repertoire.

Of note, the majority of studies evaluating the peptide sensitivity of KIR:HLA-I interactions to date have relied on external labeling with peptides, but overall, the abundance of viral peptides eluted from HLA-I compared to self-peptides is low (133, 134). Yet, antagonist peptides (i.e., peptides that are presented by HLA-I but abrogate KIR binding) can significantly interfere with KIR clustering at immune synapses and override NK-cell inhibition (135, 136). Therefore, HIV-1 infection may lead to NK-cell activation by causing a shift between antagonist and agonist peptides. Consequently, further investigations on how HIV-1 infection changes the HLA-I–presented peptide repertoire and how this impacts NK-cell function are needed. Nonetheless, CTL pressure on viral sequence appears to be dominant, as the first escape mutations arise after peak viremia and following expansion of HIV-1–specific CTLs (9). KIR are also expressed on T cells and can modulate CTL activity (75, 83); therefore, a potential role of KIR^+^ T cells in explaining *KIR/HLA* disease associations has to be considered. Overall, studies suggest a complex interplay between innate and adaptive immune pressures in driving HIV-1 sequence evolution, with HLA-I being central to the interaction with KIRs and TCRs.

### KIR3DS1 and the Non-Classical HLA-F—A Non-Classical Stress Ligand?

Genetic evidence and functional data not only implicate KIR3DS1 in HIV-1 disease but indicate a widespread effect of KIR3DS1 in autoimmunity, transplantation, cancer, and other viral infections (137). Yet, for years, a definite ligand for this receptor that could account for these effects remained elusive. Only recently, we and others discovered that KIR3DS1 can bind open conformers (OCs) of HLA-F, a non-classical HLA-I molecule (138, 139). This was confirmed *via* surface plasmon resonance (SPR), pull-down experiments, HLA-F tetramer binding studies, as well as KIR3DS1^+^ reporter cell assays (138, 139). Functionally, HLA-F OCs led to degranulation of KIR3DS1^+^ NK cells, as well as cytokine production in response to HLA-F (138). HLA-F is a non-classical HLA-I molecule with a unique combination of features. It is (i) highly conserved with one dominant allele (140) [similar to *KIR3DS1* (71)], (ii) displays tight tissue specific regulation, with a mostly intracellular localization (141, 142), and (iii) is expressed on the cell surface of activated lymphocytes (143). HLA-F is known to bind to inhibitory KIR3DL2 (138, 144), as well as inhibitory LILRBs (145, 146), whereas the results on binding of HLA-F to KIRDS4 are conflicting (138, 139, 144, 146). In HLA-F, 5 of 10 residues, which are highly conserved in other HLA class I molecules, are substituted, resulting in an altered peptide groove (146). To date, there has been no structural data published describing the OC of HLA-F. Given that KIR3DS1, KIR3DL2 and KIR3DL1 (albeit weaker), bind to HLA-F, one could imagine a role of the D0 domain in contacting HLA-F OC as the D0 domain enhances KIR3DL binding to HLA class I and mediates binding to a non-HLA class I ligand (72, 147). Nevertheless, the contact residues of KIR3DS1 to OCs of HLA-F conferring specificity and high-affinity of the interaction are entirely unknown to date. SPR data suggest that KIR3DS1 additionally binds to OCs of classical HLA class I, but so far, the functionality of this binding remains to be demonstrated (139).

We previously demonstrated that HIV-1 infection causes upregulation of HLA-F at a transcriptional level in stimulated CD4^+^ T cells. Therefore, KIR3DS1 binding to HLA-F expressed as a “stressed self” signal on HIV-1–infected cells might explain the superior ability of KIR3DS1^+^ NK cells to inhibit viral replication in autologous CD4^+^ T cells (92, 138). Thus, the interaction between KIR3DS1 and HLA-F upregulated on HIV-1–infected cells may have similarities to the well-reported upregulation of stress ligands such as ULPBs and MIC-A/MIC-B in HIV-1 infection, which are in turn recognized by the activating NK-cell receptor NKG2D (43).

**Figure 1:**
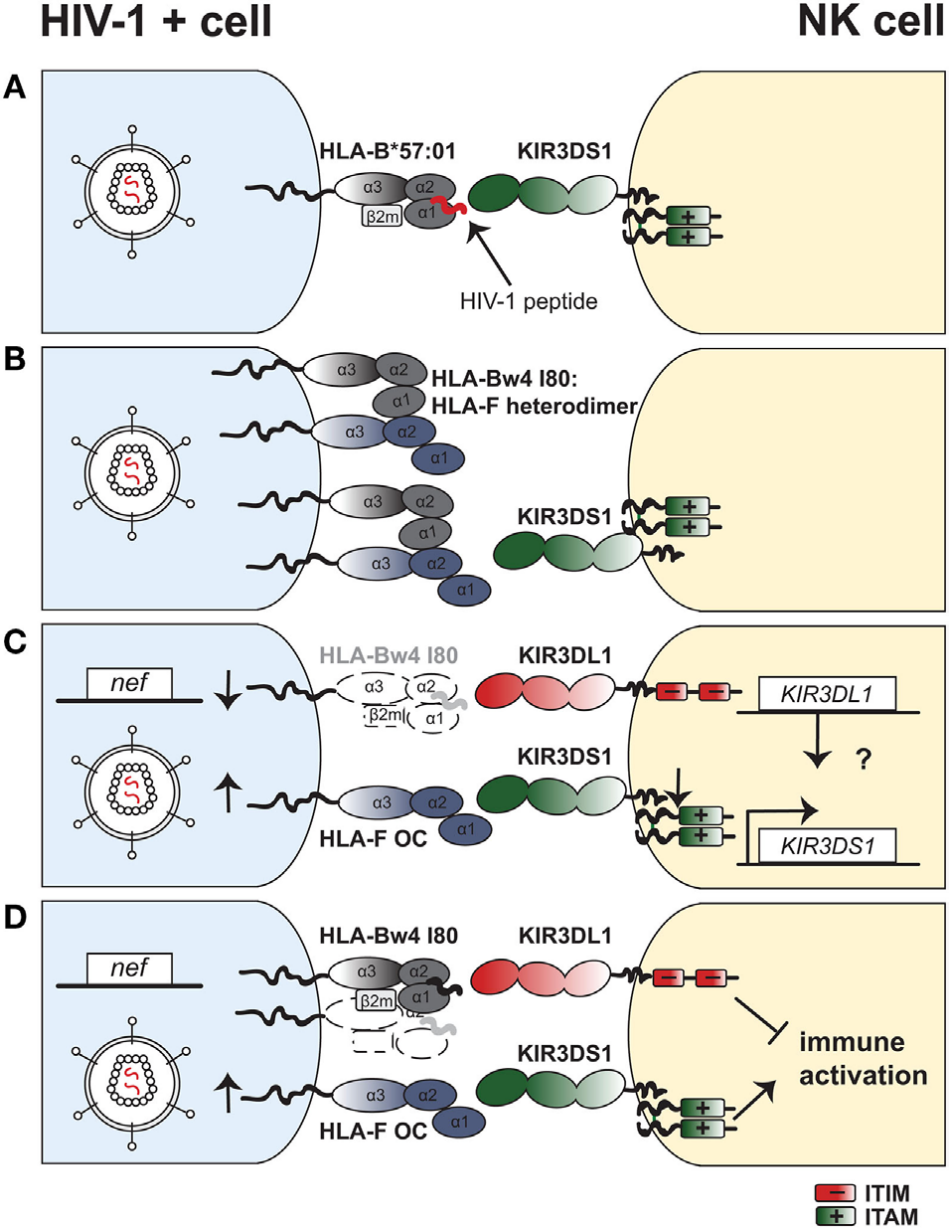
Four models with potential mechanisms to explain the underlying protective effect of the combined *KIR3DS1*–HLA-Bw4 genotype in HIV-1 infection. **(A)** Viral peptides allow for KIR3DS1 binding to HLA-B*57:01 and trigger natural killer (NK)-cell activation. Presentation of viral peptides (in red) on HLA-B*57:01 upon HIV-1 infection of target cells (blue) enables binding of KIR3DS1 on NK cells (yellow). The short cytoplasmic tail of KIR3DS1 associates to the adaptor molecule DAP12, which bears two ITAMs. **(B)** HLA-Bw4 enhances human leukocyte antigen F (HLA-F) expression at the cell surface of HIV-1–infected cells. Open conformers of HLA-F exist as heterodimers with open conformers of HLA-Bw4 on the cell surface of HIV-1–infected cells. This enhances binding and triggering *via* KIR3DS1 on NK cells. **(C)** KIR3DS1:HLA-F and KIR3DL1:HLA-Bw4 interactions have independent but synergistic protective effects in HIV-1 infection. HIV-1 infection of target cells leads to downregulation of HLA-Bw4 from the cell surface *via* action of the accessory protein Nef. Loss of HLA-Bw4 on the infected cells leads to loss of inhibition *via* KIR3DL1. Simultaneously, cell stress induced by HIV-1 infection leads to upregulation of open conformers of HLA-F, which bind to KIR3DS1 and trigger NK-cell activation. A potential epistatic regulation of *KIR3DS1* gene expression *via* the *KIR3DL1* gene is depicted. **(D)** KIR3DL1:HLA-Bw4^I80^ interactions limit KIR3DS1:HLA-F-mediated immune activation. HIV-1 infection directly (and indirectly) causes cellular stress, which in turn upregulates surface expression of HLA-F open conformers on CD4^+^ T cells and other cell types. OCs of HLA-F bind to KIR3DS1 and trigger NK-cell activation. On the other hand, KIR3DL1 binds to HLA-Bw4 molecules, which are present on HIV-1–infected cells, although at low levels due to HIV-1 Nef-mediated downregulation. Inhibitory signaling *via* KIR3DL1 limits NK-cell activation and inflammatory cytokine production, thus limiting activation *via* KIR3DS1.

Given the identification of HLA-F as a KIR3DS1 ligand, the following question remains unsolved: why is the strong genetic protective effect of *KIR3DS1* observed preferentially in combination with HLA-Bw4^I80^ in HIV-I infection? We can conceive four potential models that are not mutually exclusive and may explain this phenomenon (Figure 1):

(A) KIR3DS1 binds to HLA-B*57:01 expressing particular HIV-1 peptides: Only six residues differ in the extracellular domain of KIR3DS1 and KIR3DL1 (*013 versus *001 allele products, respectively) and one of these substitutions (L166R) abolishes binding to HLA-B*57:01. Nonetheless, one recent modeling study identified two HIV-1 derived peptides that can overcome the steric hindrance of R166 with HLA-B*57:01 R83 and allow for binding of KIR3DS1 to the HLA-B*57:01–peptide complex. Binding was of sufficient avidity to activate KIR3DS1^+^ Jurkat reporter cells (148). Thus, a change in the peptide repertoire resulting from HIV-1 infection might therefore allow KIR3DS1 to engage certain HLA-Bw4^I80^ molecules and trigger KIR3DS1^+^ NK-cell cytotoxicity. However, further studies are needed to confirm this and assess the functional relevance in natural HIV-1 infection.
(B) *HLA-Bw4^I80^enhances HLA-F expression at the cell surface of HIV-1–infected cells*: HLA-I gene products differ in their ability to form homodimers on the cell surface. In particular, the protective HLA-B*27:05 allotype is commonly expressed as a β_2_m-free disulfide-bonded homodimer (149, 150). Formation of HLA-I dimers in turn can affect recognition by immune receptors (151–153). HLA-F was reported to bind to OCs of other HLA-I to varying degrees and form heterodimers (154). Goodridge et al. discuss that the varying potential of different HLA-I gene products to interact as OCs with HLA-F may modulate HLA-F surface expression levels (144). Protective HLA-B allotypes (e.g., HLA-B*57:01) indeed demonstrate a higher degree of tapasin-dependent assembly and less stability as an OC compared to HLA-B allotypes associated with rapid progression (e.g., HLA-B*35: 03) (155). Thus, protective allotypes might differ from susceptible allotypes in their ability to interact as HLA-I OCs with HLA-F in a setting of HIV-1 infection, in turn enhancing or diminishing recognition by KIR3DS1^+^ NK cells. This would indicate a KIR3DS1: HLA-Bw4^I80^:HLA-F protective axis in HIV-1 infection that is independent of KIR3DL1.
(C) *KIR3DS1:HLA-F and KIR3DL1:HLA-Bw4^I80^interactions are independently, but synergistically protective*: Martin et al. identified the protective effect of combined *KIR3DS1* and HLA-Bw4^I80^, but the vast majority of individuals bearing *KIR3DS1* in this study were heterozygous and thus also encoded for *KIR3DL1* (22). Furthermore, Jiang et al. demonstrated that *KIR3DS1/KIR3DL1* heterozygosity in HLA-Bw4^I80^–carrying individuals conferred superior HIV-1 disease control. Therefore, it might be the heterozygous state of *KIR3DS1/KIR3DL1* in the context of HLA-Bw4^I80^ that confers protection in HIV-1 infection, rather than *KIR3DS1* alone with HLA-Bw4^I80^ (91). Thus, protection could derive from a synergistic but independent effect of KIR3DS1–HLA-F and KIR3DL1–HLA-Bw4^I80^ interactions. Long et al. showed that possessing *KIR3DS1* confers greater NK-cell functionality, also in absence of HLA-Bw4^I80^ (93). Under this model, the most effective NK cells against HIV-1–infected target cells would express both KIR3DS1 and KIR3DL1 and undergo activation *via* KIR3DS1-mediated engagement of HLA-F and KIR3DL1-dependent loss of inhibition due to HLA-B downregulation.
  Yet, there is evidence that KIR3DS1 expression and function is not completely independent from KIR3DL1, as KIR3DS1 mRNA, and KIR3DS1^+^ NK-cell frequency increases with more gene copies of *KIR3DL1* (90). Additionally, *KIR3DS1/KIR3DL1* individuals display superior viral inhibition activity than individuals with either KIR alone in the presence of HLA-Bw4 (90). Thus, there is a possibility of KIR3DL1-mediated epistatic regulation of KIR3DS1 expression and function. However, the existence of a KIR3DS1^+^KIR3DL1^+^ coexpressing NK-cell subset has not yet been definitively proven due to the limitations of current anti-KIR antibody cross-reactivity.
(D) *KIR3DL1:HLA-Bw4^I80^interactions are necessary to limit KIR3DS1-HLA-F-mediated immune activation*: As chronic viral infections can drive inflammatory processes resulting from persistent immune activation (156), downmodulation of the immune response is important for host homeostasis and preventing immunopathology; especially in HIV-1 infection where immune activation can accelerate disease progression (157). Thus, it is conceivable that inhibition of NK cells *via* KIR3DL1:HLA-Bw4 interactions may be important to counteract an exuberant immune response mediated by KIR3DS1^+^ NK cells recognizing HLA-F on “stressed”/infected cells. Moreover, education through inhibitory KIRs has been shown to promote increased survival of iKIR^+^ NK cells (105). Increased survival of educated KIR3DL1^+^ NK cells might counteract chronic immune activation that can result in disease progression. In line with this, the study of Martin et al. showed that *KIR3DS1* homozygosity without HLA-Bw4^I80^ was modestly associated with rapid progression to AIDS (22). Therefore, as supported by mouse models that implicate NK cells as “rheostats” in chronic viral infections (158), combined stimulatory and inhibitory signaling may result in a tunable antiviral response that confers optimal HIV-1 disease control without causing immunopathology.

In summary, our mechanistic understanding of how protection in HIV-1 disease is mediated in the context of combined KIR3DS1 and HLA-Bw4 is still limited and requires further study. Although we focus on NK cells, a potential role for HLA-Bw4^I80^-restricted CD8^+^ T cells expressing KIR3DS1 has also to be considered (84). So far, genetic studies of disease susceptibility have been extremely resourceful in guiding our understanding of the mechanisms involved in HIV-1 control. Therefore, HIV-1 disease association studies that are able to tease out the effect of *KIR3DS1* homozygosity in the context of HLA-Bw4^I80^ would be of great utility, but will require large sample sizes.

### The Role of Peptide:HLA-F Complex

Major histocompatibility complex class I exists in two biologically relevant conformations on the cell surface: (i) as a membrane-bound heavy chain lacking peptide and β_2_-microglobulin (β_2_m) termed open conformer (OC) or (ii) as a trimeric heavy chain:β_2_m:peptide complex (159). Recently, thermal denaturation assays demonstrated that OCs of HLA-F are more stable (146) than OCs of other HLA-I gene products (160). Earlier findings assessing stability after cold treatment suggested an increased stability of HLA-F OCs compared to open conformers of classical HLA-I (141, 142). This—and the fact that no canonical peptides could be eluted from HLA-F—supported the notion that HLA-F is mainly expressed as an OC devoid of peptide (142, 154).

Recently, the crystal structure of HLA-F (in complex with β_2_m and peptide) was solved, shedding first light onto the molecular structure of HLA-F (146). Surprisingly, this work showed that HLA-F has a unique peptide-binding grove that resembles the groove of classical HLA-I but does not anchor peptides at their N-terminus, allowing for binding of longer peptides. Indeed, peptides eluted from HLA-F and characterized by mass spectrometry had an extended length distribution compared to classical HLA-I molecules, peaking at 12 amino acids and with peptides up to 30 amino acids observed. This unconventional length rather resembles the length of HLA class II-presented peptides.

Moreover, new insights into the structure and docking mode of LILRB1 interacting with the HLA-F:β_2_m:peptide complex were gained. The LILR family (also termed LIR, ILT, or CD85) are encoded on chromosome 19 within the leukocyte receptor complex along with the KIR locus. In total, 13 different LILRs have been identified. Similar to KIRs, LILRs can provide an either inhibitory (LILRB) or activating (LILRA) signal, depending on the presence of an ITIM or the association to ITAM-containing adaptor molecules, but also depending on the cellular context (161). LILRB2 is not expressed on NK cells and its implications in HIV-1 disease are reviewed elsewhere (24). LILRB1 recognizes most classical and non-classical HLA-I molecules, except for HLA-E (162–164), given that it binds to the conserved α3 domain of the HLA-I heavy chain as well as β_2_m (165, 166). Intriguingly, the affinity of LILRB1 to peptide-bound HLA-F:β_2_m is the highest observed so far compared to other HLA-I ligands (146, 167). LILRB1 is expressed on NK cells in varying percentages (0–50% with high interindividual variability), as well as on T cells and professional antigen presenting cells such as DCs, monocytes/macrophages, and B cells (146, 168). Engagement of LILRB1 *in vitro* leads to inhibition of cytotoxicity and cytokine production in a subset of NK cells (151, 169, 170), but interestingly, LILRB1^+^ (but not LILRB1^–^) NK cells are able to markedly suppress HIV-1 replication in infected monocyte-derived DCs in a manner independent of classical HLA-I (171), hinting at a possible role of HLA-F.

**Figure 2:**
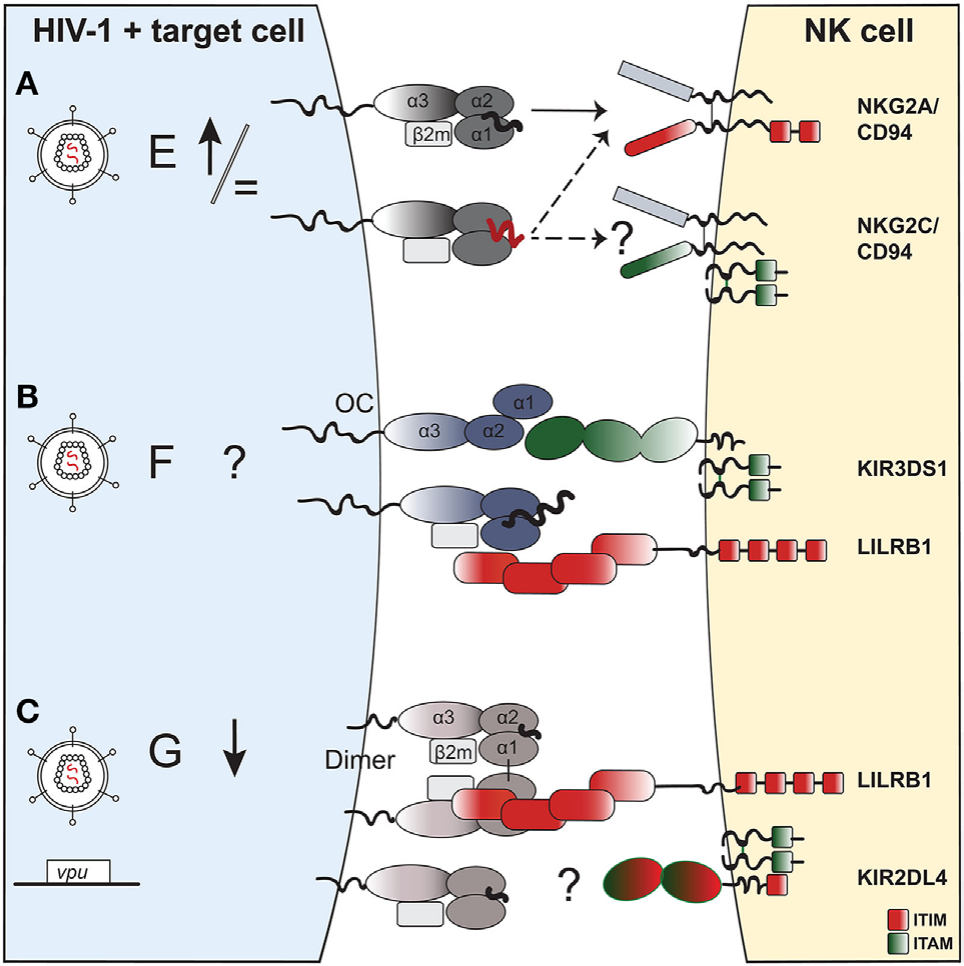
The potential impact of HIV-1 infection on expression of non-classical human leukocyte antigen class I (HLA-I) molecules on a target cell and effect on natural killer (NK)-cell receptor binding. **(A)** *HLA-E*. HIV-1 infection of target cells leaves HLA-E surface levels either unchanged or slightly upregulated. HLA-E can present self-peptides (in black) that bind to the NKG2A:CD94 receptor complex, and inhibit NK-cell function. In the context of HIV-1 infection, HLA-E can present viral or “stress”-self-peptides (in red) that abrogate (or reinforce) binding to NKG2A:CD94 and modulate NK-cell activity. The potential role of viral or “stress” peptides presented on HLA-E that could trigger the activating NKG2C:CD94 receptor complex on NK cells is currently unknown. NKG2C associates to DAP12, an adaptor molecule containing two ITAMs. **(B)** *HLA-F*. The exact impact of HIV-1 infection on HLA-F surface expression in different cell types needs yet to be established. In general, HLA-F is expressed on activated or stressed cells, potentially in distinct functionally relevant conformations: (i) an open conformer that binds to the activating NK-cell receptor KIR3DS1 or (ii) a β_2_m-bound complex presenting peptides of unusually long length for HLA-I, which allows binding of the inhibitory receptor LILRB1. **(C)** *HLA-G*: one study showed downregulation of HLA-G in monocyte-derived macrophages, potentially *via* HIV-1 Vpu, although this has not yet been confirmed in primary cells. Moreover, the functional relevance of HLA-G downregulation in antiviral immune responses has not been established to date, although HLA-G is thought to play a predominantly immunoregulatory role given its interaction with inhibitory receptors. HLA-G can form dimers on the cell surface *via* an interchain α1 disulfide bond, which enhances recognition by inhibitory LILRB1 on NK cells. KIR2DL4 binding to HLA-G remains controversial. KIR2DL4 has a dual activating and inhibitory effect on NK cells, given that its cytoplasmic tail contains an ITIM and it associates to ITAM-bearing FcRγ.

Looking at the binding footprint of LILRB1 on HLA-F, it is improbable that the interaction is sensitive to the nature of the presented peptide—in contrast to certain KIRs. In the case of KIR3DS1, it was shown that KIR3DS1^+^ reporter cells responded to HLA-F OCs, but were not triggered by peptide-bound HLA-F complexes (146). This could be due to peptide-induced conformational changes in HLA-F structure or direct steric inhibition by the bound peptides. Furthermore, inhibitory KIR3DL2 recognizes OCs of HLA-F or HLA-I and posssibly heterodimers of HLA-F with HLA-I heavy chains, with the latter also being increasingly expressed on activated lymphocytes (144). This raises interesting possibilities for a cell-stress induced conformational change in HLA-F allowing binding to activating receptors, such as KIR3DS1, while abrogating binding to inhibitory receptors, such as LILRB1. Thus, although HLA-F is not expressed on the surface of lymphocytes in a resting state (143), it potentially can exist in various conformations on stressed cells (154) with differential impact on NK-cell function (Figure 2).

### The Non-Classical HLA-G—An Immune Modulator?

Human leukocyte antigen-G is a non-classical HLA-I that displays a high degree of tissue restriction. It was first discovered in extravillous trophoblast cells in the fetal placenta (172), where HLA-G protein is abundant (173) and since then has been extensively studied in the context of reproduction. Further studies showed that under healthy conditions, HLA-G is expressed in other immune-privileged sites including the cornea (174), thymus (175), nail matrix, and on mesenchymal stem cells (176, 177). Under inflammatory conditions such as CMV infection or within a tumor microenvironment, HLA-G can be expressed on DCs and monocytes/macrophages (178, 179), and is reported to be upregulated in monocytes treated with IFN-γ (a potent inducer of HLA-I and -II expression) as well as IL-10 (180–182). Also, an increasing number of studies shows aberrant HLA-G mRNA expression by tumors (183, 184) [reviewed in Ref. (185)], but some of these findings remain controversial as in other studies no HLA-G protein was identified (179, 186) [reviewed in Ref. (187)]. Overall, there is evidence that HLA-G expression is induced on various immune cells under inflammatory conditions resulting from infections, allergies, or allogeneic stimulation following transplantation (188–191).

As a result of a premature stop codon in exon 6 (192), the cytoplasmic tail of HLA-G is truncated and the heavy chain has a molecular weight of only 39 kDa, compared to the 45 kDa weight of classical HLA class I heavy chain. In total, seven splicing variants of HLA-G have been described (193, 194). The predominant splice variant *in vivo* is HLA-G1, which encodes for the full-length, membrane-bound HLA-G protein (195, 196). Alternatively, soluble HLA-G (sHLA-G) can be generated from three splice variants or *via* proteolysis of the HLA-G1 isoform (197). Interestingly, sHLA-G can confer a protective effect to cells normally permissive to NK-cell killing (197). Apart from HLA-G1, three other alternatively spliced transcripts encode membrane-bound HLA-G, albeit in a truncated form: HLA-G2 lacks the α2 domain, HLA-G4 lacks the α3 domain, and HLA-G3 lacks both the α2 and α3 domains (198). These transcripts were reported to inhibit NK-cell function, although it remains unclear through which NK-cell receptors this occurs (198–200). Moreover, it was suggested that isoforms HLA-G2 and -G3 are expressed in individuals homozygous for the HLA-G*0105N null allele (201), possibly explaining the existence of healthy adults lacking full length HLA-G1 (201). Of note, all splicing variants encode the leader sequence enabling HLA-E expression (202) and thereby their expression in target cells can indirectly inhibit NK cells *via* NKG2A:CD94 (163).

To date, 18 distinct functional proteins of HLA-G have been identified, with the *HLA-G* gene encoding a total of 54 *HLA-G* alleles (including two *HLA-G* null alleles) (56). While most of the polymorphism of classical HLA-I genes lies in the α1 and α2 domains that bear the peptide-binding groove (203), HLA-G has a relatively conserved peptide-binding groove and has allelic variability occurring within the 3′UTR, which is important for posttranscriptional regulation of HLA-G (203, 204). Peptides eluted from HLA-G thus far appear to be derived from a restricted number of proteins (205) and a crystal structure demonstrates that these presented peptides are buried deep within the peptide-binding groove (206). The induction of an HLA-G-restricted CD8^+^ T cell response against a human cytomegalovirus peptide in mice was described, but the cytolytic capacity of these T cells was limited (207). Overall, it seems that the immune modulatory functions of HLA-G mediated through binding of inhibitory receptors expressed on a variety of immune cells dominates over a potential role in presenting peptides.

### NK-Cell Receptors Recognizing HLA-G

Human leukocyte antigen-G is recognized by LILRs with greater affinity than HLA-A, -B, or -C molecules (208). In addition, HLA-G is unique in possessing a cysteine at position 42 of its α1 domain, which allows for an unusual conformation of HLA-G as a homodimer of two β_2_m-associated HLA-G complexes (152, 209, 210) (Figure 2C). This conformation dramatically enhances recognition and signaling of LILRB1 (151) and has been demonstrated to occur naturally on trophoblasts (173). Indeed, inhibition of LILRB1^+^ NK-cell function is sensitive to the conformation of HLA-G, as the heavy chain of HLA-G alone does not inhibit LILRB1^+^ NK cells (211). Studies measuring inhibition of LILRB1^+^ NK-cell cytolytic function *via* HLA-G have to account for HLA-E expression as it is upregulated through the HLA-G leader peptide—an exception being the K562 cell line, which does not express HLA-E (212). Independent of HLA-E, HLA-G interferes with immunological synapse formation and inhibits NK-cell cytotoxicity (212, 213). Additionally, Riteau et al. demonstrated that HLA-G expression has a major inhibitory effect on NK cell lysis through LILRB1, also when coexpressed with other HLA-I ligands (214). Besides the inhibitory effect of HLA-G expression on NK-cell-effector function itself, HLA-G can impair NK-DC crosstalk. Pretreatment of DCs with sHLA-G leads to reduced activation and IFN-γ production by NK cells (215), while IFN-γ in turn triggers HLA-G surface expression (180, 181). This again supports the notion that HLA-G has tolerogenic properties.

In addition to LILRB1, HLA-G has been proposed to modulate NK-cell function *via* binding KIR2DL4. *KIR2DL4* is a framework gene within the KIR locus and thus is present in virtually all haplotypes, but there is a high frequency of alleles lacking the transmembrane domain or having truncated cytoplasmic tails (216). In peripheral blood, expression of KIR2DL4 is weak and restricted to the CD56^bright^ subset, but can be induced on NK cells *in vitro* with stimulation (217, 218). KIR2DL4 has unique functional properties compared to other receptors of this family. A positively basic arginine residue in the transmembrane domain allows for association with the activating Fc receptor gamma protein (219), while the long cytoplasmic tail contains one immunoreceptor tyrosine-based inhibitory motif (ITIM). This results in mixed activating and inhibitory signaling, which has been shown to occur *in vitro* (219–222). In line with this, crosslinking of KIR2DL4 on peripheral blood NK cells induces IFN-γ production, and (albeit weaker) NK-cell cytotoxicity (217, 218, 223).

Newer reports provide conflicting evidence regarding the interaction of HLA-G with KIR2DL4 (224, 225). Although several groups reported binding using various techniques including cellular transfectants, SPR, and functional assays (169, 226–230), others have failed to reproduce KIR2DL4 binding *via* SPR, tetramers, or functional IFN-γ responses to sHLA-G (210, 231, 232). The crystal structure of the extracellular domains of KIR2DL4 solved by Moradi et al. (233) demonstrated oligomerization of KIR2DL4, uncharacteristic of other KIRs. In this study, no binding of KIR2DL4 to HLA-G was detected *via* SPR (233). An explanation might be that signaling *via* KIR2DL4 only occurs upon concentration of the ligand in endosomes [as discussed in Ref. (187)], since sHLA-G endocytosed into KIR2DL4-containing compartments was shown to induce cytokine secretion of NK cells (229, 234). Regardless of its binding to HLA-G, higher copy numbers of KIR2DL4 have been linked to better survival of CD4^+^ T cells and increased IFN-γ responses from NK cells during acute SIV infection in rhesus macaques (235).

### HLA-G Expression in HIV-1

Only a low percentage of immune cells in healthy subjects expresses HLA-G, whereas in HIV-1 infection a substantial upregulation of HLA-G has been observed in both peripheral blood monocytes and T-cell subsets (236). This was later partly attributed to antiretroviral treatment, as frequencies of HLA-G^+^ monocytes decreased after treatment interruption (237). In fact, nucleoside reverse transcriptase inhibitors were found to increase HLA-G expression, whereas protease inhibitors did not (238). A role for HLA-G^+^ HIV-1–restricted CD8^+^ T cells has furthermore been described in HIV-1–infected subjects (239). Contrary to *in vivo* studies of high HLA-G expression levels on monocytes of patients undergoing HAART (236–238), one study showed downregulation of HLA-G1 surface expression in HIV-1–infected monocyte-derived macrophages *in vitro* (240). This downregulation was suggested to be mediated *via* HIV-1 Vpu (240), given that the truncated tail of HLA-G renders it resistant to HIV-1 Nef-mediated downregulation (241). Yet, this needs to be confirmed in primary cells. Overall, how HIV-1 directly impacts HLA-G expression in different cell types remains unclear.

In addition to inhibiting DC function *via* LILRB2 (242) and regulating CD4^+^ T-cell proliferation (243), sHLA-G can inhibit NK-cell killing *in vitro* and may therefore suppress NK-cell function *in vivo* (197). sHLA-G plasma levels change during the course of HIV-1 infection and treatment, as two groups reported high sHLA-G levels in early infection (244) with a significant decrease after treatment initiation (245). In rapid progressors, however, levels of sHLA-G were persistently elevated even despite treatment initiation, while this was not the case for untreated normal progressors and long-term non-progressors (244). Furthermore, sHLA-G levels were higher in patients with opportunistic infections, indicating a potential role of sHLA-G as a surrogate marker of disease progression (246). In a cohort of female commercial sex workers from Benin, HIV-1–infected subjects were reported to have lower levels of sHLA-G in plasma (247) but higher levels of sHLA-G in the genital mucosa (248). Of note, levels of sHLA-G are also in part determined genetically by distinct *HLA-G* alleles (249). Thus, data on sHLA-G levels in HIV-1 infection need to be carefully controlled for confounding factors such as HAART (237, 238), *HLA-G* genetic background (249), sampling site (247, 248), or coinfections (246, 248). In summary, it is not known whether higher sHLA-G levels have direct functional consequences on HIV-1 disease progression *via* modulation of NK and other immune cells, or whether sHLA-G levels are rather a reflection of viremia and the antiviral immune response.

### Genetic Evidence for a Role of HLA-G in HIV-1 Infection

Although HLA-G polymorphisms are limited, certain HLA-G alleles have been suggested to be involved in susceptibility to HIV-1 infection. In 2004, Matte et al. reported that the HLA-G*0105N allele, a null variant which does not encode functional HLA-G1, was protective in HIV-1 acquisition, whereas the HLA-G*01:01:08 allele encoding for full-length HLA-G increased the risk of HIV-1 infection. They formulated the hypothesis that non-functional HLA-G proteins may allow for better NK-cell killing of HIV-1–infected cells (250). This observation was not consistent with findings of subsequent studies, which reported either enrichment of HLA-G*0105N in HIV-1–positive women (251) or did not identify HLA-G*0105N allele as a disease modifying factor (252). Other *HLA-G* alleles identified were HLA-G*01:04:04, which associated with susceptibility to HIV-1 infection, and HLA-G*01:01:01, which was enriched in HIV-1–resistant women (252). One study states that these conflicting findings may be explained by variation of *HLA-G* polymorphisms among different ethnic populations and reports no association of *HLA-G* polymorphisms to HIV-1 susceptibility except in African-American cohorts (253).

As HLA-G is an important player involved in maternal-fetal tolerance, *HLA-G* polymorphisms have been studied in the context of vertical HIV-1 transmission from mother-to-child. Mothers bearing the HLA-G*01:03 allele were less likely to perinatally transmit HIV-1 (254). Upregulation of the functional isoform HLA-G1 mRNA in the placenta has been associated with increased risk of HIV-1 mother-to-child transmission (255). Further studies have assessed the risk of variants in the 5′ and 3′UTR of HLA-G, and in particular, the impact of the 14-bp insertion/deletion in the 3′UTR of HLA-G on mother-to-child transmission. In healthy subjects, the 14-bp insertion genotype (ins/ins) correlates with lower plasma levels of sHLA-G (256). *In vitro*, transfection of the 14-bp ins/ins HLA-G into K562 cells resulted in increased levels of membrane-bound HLA-G1 expression with higher mRNA stability and lower sHLA-G1 ratio (257). However, studies on the impact of the 14-bp insertion on HIV-1 vertical transmission risk report conflicting results (258–260). In horizontal transmission, the frequency of the 14-bp ins/ins genotype was enriched in HIV-1–infected patients in African (but not European) subjects (261). Overall, population studies attempting to shed light on the question whether functional versus non-functional *HLA-G* alleles are associated with HIV-1 susceptibility have painted an inconsistent picture. Moreover, posttranscriptional regulation of the *HLA-G* gene through variations in the 3′ and 5′ LTR and alternative splicing has to be considered as an important genetic factor modulating HLA-G expression levels in these studies.

### The Oligomorphic Interaction between HLA-E and NKG2:CD94—A Contrast to the Diversified HLA-KIR System

Inhibition of NK cells can be achieved either through highly diversified KIR:HLA-I interactions or through a second inhibitory system indirectly monitoring the level of overall HLA-I expression. This latter inhibitory mechanism is achieved *via* the well-conserved NK-cell receptor–ligand interaction of NKG2A/CD94 with HLA-E (262). Contrary to other non-classical HLA-I gene products, HLA-E is ubiquitously expressed (263), but at substantially lower levels as compared to classical HLA-A, -B, and -C (264). Its expression is dependent on the expression of other HLA-I, as it presents a nonamer peptide derived from the signal sequence of several HLA-A, -B, and -C gene products as well as HLA-G. HLA-F and HLA-E itself lack an HLA-E–presented leader peptide (265).

Human leukocyte antigen-E has restricted polymorphism with to date only 25 known alleles (56), of which two—HLA-E*01:01 and *01:03—are the most frequent in the human population and are believed to be in balancing selection (266, 267). HLA-E*01:01 encodes for an arginine at position 107 (HLA-E^R^), whereas HLA-E*01:03 encodes for a glycine at this position (HLA-E^G^). This substitution leads to higher surface expression levels of the latter, despite similar intracellular protein levels (160). HLA-E is highly relevant to innate immune responses due to its interaction with heterodimeric NKG2/CD94 type II transmembrane-anchored receptors, which are expressed on a large proportion of NK cells as well as on a subset of CD4^+^ and CD8^+^ T cells (268–270).

Natural killer group 2 receptors are a family of C-type lectin receptors encoded within the NK gene complex on chromosome 12p12-13 (271). Almost all NKG2 gene products heterodimerize with CD94, a non-signaling invariant glycoprotein also encoded within the NK gene complex. These include *NKG2A* [which produces NKG2A and NKG2B gene products *via* alternative splicing (272)], *NKG2C, NKG2E, NKG2F*, and *NKG2H*. The *NKG2D* gene is also located within the NK gene complex, but its gene product has low sequence homology to other NKG2 receptors and forms an NKG2D:NKG2D homodimer (without CD94) that binds to the stress ligands MIC-A, MIC-B, and ULBPs, but not to HLA-E (273). Unlike *KIR* genes, *NKG2* genes exhibit limited polymorphism (262, 274). Aside from being expressed widely on NK cells, they can also be expressed on subsets of T cells (275). Here, we focus on NKG2A:CD94 and NKG2C:CD94 receptor complexes, both of which bind HLA-E but have opposite effects on NK-cell function. While NKG2A signaling inhibits NK-cell cytotoxicity *via* two ITIMs in its cytoplasmic tail (276), NKG2C delivers activating signals through its associated adaptor molecule DAP12 (277).

Despite their similarity, the two major alleles of HLA-E differ in the subset of peptides they present (278). An example is the HLA-B*27–derived leader peptide, which stabilizes HLA-E^G^, but does not bind detectably to HLA-E^R^ (160, 279). Similar to KIR:HLA interactions, binding of the NKG2:CD94 heterodimer to HLA-E is sensitive to the presented peptide (279, 280). The crystal structures of NKG2A:CD94 and NKG2C:CD94 receptor complexes bound to HLA-E presenting the HLA-G leader peptide (VMAPRTLFL; VL9) illustrate that both subunits (NKG2 and CD94) intimately interact with the peptide-binding domains (α1 and α2) of HLA-E. Interestingly, CD94 occupied the majority of the binding site, yet despite this, the NKG2A:CD94 complex had six times stronger binding affinity to HLA-E:VL9 than NKG2C:CD94 (280). Consequently, it is believed that CD94 is the main driver of HLA-E binding and peptide sensitivity, while the NKG2 subunit modulates affinity (and possibly sensitivity to some extent). Leader peptides of classical HLA-I presented on HLA-E do not trigger NK-cell activation through NKG2C, whereas NKG2A^+^ NK cells are potently inhibited by a wide range of different HLA-I–derived leader peptides (281). Therefore, the NKG2A:CD94–HLA-E interaction allows NK cells to indirectly monitor for changes in overall HLA-I expression without causing aberrant immune activation through NKG2C:CD94. An exception to this is HLA-E in complex with the HLA-G leader peptide, which can engage NKG2C:CD94 and trigger activation (279, 281). As HLA-G displays high tissue-specific restriction, this nonetheless allows for tight regulation of NKG2C triggering. The amount of surface stabilization of HLA-E by various leader peptides does not strictly correlate with the level of inhibition through NKG2A:CD94, which emphasizes the role of specific peptides in the binding of NKG2A:CD94 to HLA-E (282).

### Peptide Presentation by HLA-E in the Context of Viral Infections

Like classical HLA-I, HLA-E can also present virus- or “stress”-derived peptides. The leader sequences of heat shock protein 60 (HSP60), which is induced under stress conditions (283), stabilizes HLA-E, but disrupts binding to NKG2A:CD94 and thus disinhibits NK-cell function (284). HLA-E can also be the target of viral immune evasion. CMV, for example, encodes for a sequence identical to the HLA-C*03 leader peptide that can increase HLA-E expression and inhibit NK-cell cytotoxicity (285). Additionally, an HCV-derived epitope (HCV Core35-44) stabilizes HLA-E and inhibits NK-cell lysis (286). Cheent et al. showed that viral- or heat shock protein-derived peptides in isolation did not inhibit NK-cell lysis. However, these peptides enhanced inhibition in the presence of HLA-E–presented leader peptides and therefore were termed “synergistic peptides.” Confocal microscopy has shown that these synergistic peptides act by recruiting non-signaling CD94 (without NKG2A) to the immunological synapse (262). Similar to peptide antagonism in KIR-HLA interactions (135, 136), this adds an additional layer of complexity to peptide-dependent modulation of NK-cell-effector function.

For HIV-1, the capsid-derived p24 aa14–22 epitope AISPRTLNA (AA9) has been described to stabilize HLA-E. One study by Natterman et al. demonstrated that AA9 inhibited NK cell-mediated cytolysis of peptide-pulsed HLA-E–transfected K562 cells (287), and that NK-cell killing could be restored *via* antibody blockade of either HLA-E or NKG2A. Contrary to this study, however, Davis et al. reported that HLA-E:AA9 tetramers did not bind to NKG2A^+^ CD56^bright^ NK cells (while HLA-E:VL9 tetramers did). Thus, the authors suggest a potential role for the AA9 peptide in abrogating HLA-E binding to NKG2A:CD94 on NK cells, explaining enhanced degranulation of NKG2A^+^ NK cells against HIV-1–infected cells as compared to NKG2A^−^ NK cells (288). In line with a role of HLA-E in HIV-1 infection, a genetic study in a cohort of Zimbabwean women demonstrated a four-fold reduced risk of HIV-1 acquisition in individuals homozygous for HLA-E*01:03 (HLA-E^G^) alleles compared to heterozygous or HLA-E*01:01 homozygous individuals. Given that HLA-E^G^ is a high-expression allele, the authors speculated that increased presentation of HIV-1 peptides by HLA-E enhances NK cell cytotoxicity against HIV-1–infected target cells during the initial stages of infection (289).

Besides the role of HLA-E in innate immunity, increasing evidence demonstrates that HLA-E presentation of viral peptides derived from CMV, EBV, and HCV can elicit HLA-E–restricted CD8^+^ T-cell responses (290–292). Furthermore, Hansen et al. (293) showed that inoculation of rhesus CMV-based SIV_gag_ vectors leads to presentation of surprisingly diverse epitopes on MHC-E, inducing a broadly directed and protective CD8^+^ T cell response in rhesus macaques (293). So far, HIV-1–specific HLA-E–restricted CD8^+^ T cells have not been shown in humans (294), but the conserved nature of *HLA-E* alleles among different populations, its ability to present viral peptides, and its dual role in innate and adaptive immunity renders HLA-E an important target for future research.

### NKG2A^+^ NK Cells—A Subset with Enhanced (Not Reduced) Antiviral Capacity in HIV-1

Chronic HIV-1 viremia leads to a decrease in the proportion of NK cells expressing NKG2A (32, 295–297), and normal NKG2A levels are restored only after prolonged times of antiretroviral therapy (297). Subset analyses show, however, that NKG2A^+^ cell frequency increases within the CD56^dim^CD16^bright^ NK-cell subset over the course of HIV-1 disease progression, whereas NKG2A^+^ cell frequency is decreased in the dysfunctional CD56^−^ NK cell subset (298). Given that this highly dysfunctional CD56^−^ NK cell subset with poor cytotoxic capacity expands in viremic subjects (33, 34), bulk NKG2A^+^ NK-cell frequencies are reduced (298). Presence of viremia in patients with low CD4^+^ T-cell counts correlated with significantly higher NKG2A^+^ frequencies on CD56^dim^CD16^bright^ NK cells compared to aviremic patients with low CD4^+^ T cell counts (298), which may suggest a potential effect of long-term HIV-1 exposure itself on modulating NKG2A expression.

On the other side of the equation, HLA-E levels on CD4^+^ T cells from HIV-1–infected patients increase with declining CD4^+^ T cell counts *in vivo* (299). Upon HIV-1 infection or reactivation *in vitro*, HLA-E surface levels remain unchanged (36, 114, 288) or increase (287, 299). Functionally, blocking of the inhibitory NKG2A:CD94 interaction with HLA-E increases the ability of NK cells to kill HIV-1–infected CD4^+^ T cells *in vitro* (114, 287). Similarly, blocking of NKG2A enhances ADCC of NK cells toward antibody-coated HIV-1–infected CD4^+^ T cell blasts (115). Although these initial data implied the notion that HLA-E–NKG2A:CD94 interactions were inhibitory and detrimental to elimination of HIV-1–infected cells, recent experimental data demonstrated a superior ability of the NKG2A^+^ NK-cell subset to degranulate in response to HIV-1–infected CD4^+^ T-cell blast compared to NKG2A^−^ subsets (288). Moreover, NKG2A^+^ NK cells showed the highest polyfunctional responses with increased IFN-γ and MIP-1β, as well as higher CD107a expression against HIV-1–infected CD4^+^ T cell blasts (300). This suggests that HLA-E-mediated inhibition of NK-cell function *via* engagement of NKG2A:CD94 is incomplete, potentially due to a skewed peptide repertoire in infected cells (288) (Figure 2A). Moreover, the increased functionality of NKG2A^+^ NK cells highlights the concept that the inhibitory NKG2A:CD94 receptor is important in NK-cell education (301), as described later in more detail. Taken together, the factors driving an overall decline in NK-cell function in HIV-1–infected individuals are not entirely clear, although decreased frequency of NKG2A^+^ NK cells may play a role.

### NKG2C^+^ NK Cells—A Role in HIV-1 Independent (or Dependent) of CMV?

It is conceivable that ligation of activating NKG2C:CD94 *via* HLA-E may enhance cytotoxicity toward HIV-1–infected cells, but this has not been demonstrated. In healthy subjects, NKG2C is expressed only at low-to-moderate frequencies depending on *NKG2C* zygosity and CMV status (288, 302). In HIV-1–infected subjects, an increased frequency of NKG2C^+^ NK cells can be detected (295), independent of HIV-1 disease stage or presence of viremia (298), leading to a reversed NKG2A^+^-to-NKG2C^+^ NK-cell ratio in HIV-1–infected subjects compared to healthy controls (296). Additionally, NKG2C^+^ NK cells form part of the dysfunctional CD56^−^CD16^+^ NK-cell population in HIV-1–positive viremic patients (303). Thus, NKG2C expression appears to be modulated by HIV-1 infection, but differences in NKG2C^+^ NK-cell activity toward HIV-1–infected cells have not been demonstrated (Figure 2A).

It is important to note that CMV infection substantially skews the NK-cell repertoire toward NKG2C-expressing NK cells (304, 305). Furthermore, NK cells of CMV seropositive patients display enhanced cytotoxicity against target cells expressing HLA-E, which can be blocked by anti-NKG2C (306). Therefore, coinfection of CMV in HIV-1–infected patients is a highly relevant confounding factor when assessing NKG2C^+^ frequencies and function on NK cells. In a cohort of HIV-1–positive aviremic individuals, the association between increased NKG2C expression and HIV-1 infection disappeared when accounting for CMV seropositivity (307). Furthermore, Brunetta et al. showed that NKG2C^+^ NK-cell frequencies are higher in CMV seropositive individuals with HIV-1 infection compared to CMV seropositive HIV-1–negative subjects (297). Overall, the leading notion is that HIV-1 infection may render individuals more susceptible to CMV reactivation and impair immune control of CMV, potentially explaining the higher degree of CMV-driven expansion of NKG2C^+^ NK-cell subsets in HIV-1–infected subjects (308, 309). Additional evidence for a potential role of NKG2C comes from HIV-1 disease association studies, where homozygous deletion of *NKG2C* in a cohort of HIV-1–infected subjects was associated with increased risk of HIV-1 infection. Moreover, a genotype with two functional copies of *NKG2C* was significantly enriched in long-term non-progressors compared to normal progressors. This indicates a functional role for the NKG2C receptor in HIV-1 infection (310), which remains to be established experimentally.

CMV-driven expansion of NKG2C^+^ NK cells has received great interest as it has been implied in conferring adaptive, memory-like functions to NK cells (311). Briefly, first evidence came from a study in hematopoietic stem cell transplantation (HSCT), where infusing NK cells from CMV-seropositive donors into CMV-seropositive HSCT recipients led to expansion of donor NKG2C^+^ NK cells and production of increased amounts of IFN-γ in comparison to donor NKG2C^+^ NK cells infused into CMV seronegative HSCT recipients (312). This hinted at a previous priming of donor NKG2C^+^ NK cells leading to an enhanced antiviral response upon re-challenge with CMV in the CMV seropositive HSCT recipient (312). Additional evidence of adaptive NK-cell function in a rhesus macaque model demonstrated that splenic NK cells derived from previously SIV-infected macaques specifically lysed DCs pulsed with SIV Gag or Env *in vitro*. Remarkably, antigen-specific NK-cell cytotoxicity against Gag- or Env-pulsed DCs was reduced by blocking NKG2A and NKG2C, which suggests a potential role of these receptors in NK-cell memory (313). Taken together, in humans, the role of activating NKG2C:CD94 receptors in HIV-1 infection, either for increased recognition of HIV-1–infected target cells *via* HLA-E (independent of CMV) or for a potential HIV-1 specific NK-cell response remains to be further investigated.

### HLA-E Is Affected by Dimorphism in the Leader Peptide of HLA-B

An additional factor impacting HLA-E surface expression is a dimorphism in the leader peptide of HLA-A, -B, and -C. *HLA-A* and *HLA-C* alleles encode for a methionine at position 2 of the leader sequence, whereas *HLA-B* alleles can either encode for methionine (-21M) or threonine (-21T) at this position. Leader sequences with threonine at P2 do not allow for stable induction of HLA-E surface levels and consequently fail to confer protection from NK cells through engagement of inhibitory NKG2A:CD94 (279). In HIV-1 infection, *HLA-B* alleles containing a Bw4 motif are associated with protection from AIDS (69) and all HLA-Bw4 alleles (with the exception of HLA-B*38:01) encode for the −21T polymorphism (42), whereas HLA-Bw6 alleles encode for either −21T or −21M. In a large cohort of serodiscordant Zambian couples, Merino et al. aimed to elucidate the impact of HLA-B leader peptide dimorphism independent of the Bw4 motif. Compound carriage of either Bw6/−21T or Bw4/−21T alleles displayed similar levels of protection in comparison to Bw6/−21M alleles, which were associated with increased risk of seroconversion. This indicates an independent protective effect of the −21T dimorphism on HIV-1 acquisition (314). Moreover, NK cells lysed HIV-1–infected CD4^+^ T cells or HIV-1–infected monocyte-derived macrophages preferentially when target cells encoded for −21T/T over a range of various HIV-1 strains. Antibody-mediated blockade of HLA-E on −21M/M target cells increased NK-cell cytotoxicity, whereas no change was observed for −21T/T target cells. Surprisingly, in this study mean fluorescence intensity of HLA-E surface expression did not differ between the −21T/T, T/M, or M/M subsets (315). However, recent analyses employing mass cytometry revealed that donors with at least one copy of −21M displayed increased surface HLA-E levels compared to −21T homozygous donors. NK cells of −21M donors displayed reduced amounts and frequencies of NKG2A:CD94, but a higher phenotypic diversity (42). In this study, Horowitz et al. additionally showed that the increased availability of HLA-E peptides in −21M donors is important for NK-cell functionality (42).

Shaping of NK cell function *via* self-reactive inhibitory NK-cell receptors is a well-described process termed licensing or education (316). It is governed by two independent systems, the well-conserved interaction of NKG2A with HLA-E and the diversified interaction of HLA-I and inhibitory KIR (301). The presence of −21M leader peptides available for HLA-E stabilization indeed correlated with an increased polyfunctional NK-cell response in terms of ADCC, IFN-γ production and degranulation against the missing-self K562 target cell line compared to NK cells derived from donors with a −21T/T genotype (42). Based on the dimorphism in the HLA-B leader peptide, Horowitz et al. described the evolution of two distinct HLA-I haplotypes, which can be distinguished by the inhibitory receptor system operating in NK-cell education. The first, more ancient haplotype encoding the HLA-E permissive −21M and HLA-C1 alleles is skewed toward the supply of ligands for NKG2A:CD94, whereas the second haplotype encodes the non-HLA-E–permissive −21T dimorphism, *HLA-B* with a Bw4 motif and HLA-C2/C1 allotypes, hence being more skewed toward encoding strong KIR ligands (42). Studying the two potential routes of licensing, Bernard et al. reported that the NKG2A^+^ NK cell subset mounted the highest polyfunctional response against infected CD4^+^ T cells, without further modulation through coexpression of inhibitory KIR3DL1 (300). An earlier study reported a dual effect of NKG2A and inhibitory KIR coexpression in promoting NK cell education as well as survival (105). Coexpression of inhibitory KIR (with the presence of cognate ligand) with the activating NKG2C receptor following CMV reactivation after hematopoietic cell transplantation was required for robust cytokine production by NK cells (317). This raises the question of which combination of KIR and NKG2 receptors results in best possible NK-cell functionality and survival in combating HIV-1 infection. Overall, HLA-E—aside from presenting peptides—has clearly an additional function in NK-cell education through NKG2A. This in turn may explain the antiviral capacity of NKG2A^+^ NK cells as observed *in vitro* following HIV-1 infection.

## Concluding Remarks

Studies from the preantiretroviral treatment era suggest that early events in acute HIV-1 infection influence the rate of HIV-1 disease progression. NK cells, as first-responding innate effector cells, have been shown to expand in early HIV-1 infection and kill HIV-1–infected cells, with genetic studies robustly linking variants in NK-cell receptors to HIV-1 acquisition and disease progression. Additionally, according to mouse models of chronic viral infections, NK cells have the potential to regulate adaptive immune responses, possibly even impairing an effective adaptive response (158, 318). Notably, the antiviral effector potential of NK cells is closely linked to HLA-I. HLA-I allows not only for effective NK-cell education, but also modulates NK-cell activity toward HIV-1-infected cells *via* changes in HLA-I surface expression and peptide presentation. While numerous studies have established a role for the KIR interaction with classical HLA-I in HIV-1, recent advances have increased our understanding of non-classical HLA-E, -F, and -G in HIV-1 infection. First, HLA-F was identified as a ligand for KIR3DS1, which is prominently associated with HIV-1 disease control. HLA-F may serve as a “stress” signal on HIV-1–infected cells, at best enhancing KIR3DS1^+^ NK-cell killing of infected cells, and at worst mediating HIV-1–associated immunopathology (Figure 2B). Second, HLA-E expression levels are not downregulated in HIV-1, which is important as HLA-E is capable of presenting viral peptides. Moreover, HLA-E can tune NK-cell function through NKG2A in virtually all individuals, and is linked to a superior antiviral capacity of NKG2A^+^ NK cells (Figure 2A). Third, HLA-G has predominantly immunomodulatory properties (rather than a peptide-presenting function), and although genetic studies are teasing apart the link between *HLA-G* polymorphisms and HIV-1 disease, the impact of HLA-G on NK-cell function in HIV-1 has yet to be determined (Figure 2C). Eventually, the unique properties of these non-classical HLA-I molecules and their conservation between individuals renders them an ideal target for new approaches aimed at harnessing innate immunity against HIV-1.

## Author Contributions

AH wrote the first draft of the manuscript, WG-B and MA have made substantial, direct, and intellectual contributions to the work and all authors approved it for publication.

## Conflict of Interest Statement

The authors declare that the research was conducted in the absence of any commercial or financial relationships that could be construed as a potential conflict of interest.
